# Machine learning-based construction of a ferroptosis and necroptosis associated lncRNA signature for predicting prognosis and immunotherapy response in hepatocellular cancer

**DOI:** 10.3389/fonc.2023.1171878

**Published:** 2023-04-20

**Authors:** Lei Zhao, Zhixuan You, Zhixun Bai, Jian Xie

**Affiliations:** ^1^ The Third Clinical lnstitute, Guangzhou Medical University, Guangzhou, China; ^2^ Department of Nephrology, The Second Affiliated Hospital of Zunyi Medical University, Zunyi, China; ^3^ Department of Medical Genetics, Zunyi Medical University, Zunyi, China

**Keywords:** LIHC, lncRNA, ferroptosis, necroptosis, immunotherapy

## Abstract

**Introduction:**

Liver hepatocellular carcinoma (LIHC), one of the most common malignancies worldwide, occurs with high incidence and mortality. Ferroptosis and necroptosis are critically associated with LIHC prognosis. Some long non-coding RNAs (lncRNAs) have been found to induce ferroptosis and necroptosis in hepatocellular carcinoma cells.

**Methods:**

Cox regression analysis was used to construct a risk model for LIHC based on differentially expressed ferroptosis and necroptosis related lncRNAs (F-NLRs), and their expression in SMMC7721, HepG2 and WRL68 cells was detected by qPCR.

**Results:**

Five F-NLRs were associated with LIHC prognosis, including KDM4A-AS1, ZFPM2-AS1, AC099850.3, MKLN1-AS, and BACE1-AS. Kaplan-Meier survival analysis indicated that patients with LIHC in the high-risk group were associated with poor prognosis. The combined F-NLR signature model demonstrated a prognostic AUC value of 0.789 and was more accurate than standard clinical variables for predicting LIHC prognosis. T cell functions and immunotherapy responses differed significantly between patients in the low- and high-risk groups. Additionally, immune checkpoints and m6A-related genes were differentially expressed between patients in the two risk groups. Furthermore, proteins encoded by the five F-NLRs were overexpressed in four liver cancer cell lines compared to that in human liver cell line WRL68. Pan-cancer examination revealed that expression levels of the five F-NLRs differed between most common tumor types and normal tissues.

**Conclusion:**

F-NLRs identified in this study provide a predictive signature representing ferroptosis and necroptosis in LIHC, which correlated well with patient prognosis, clinicopathological characteristics, and immunotherapy responses. The study findings help to elucidate the mechanisms of F-NLRs in LIHC and provide further guidance for the selection and development of immunotherapeutic agents for LIHC.

## Introduction

Liver hepatocellular carcinoma (LIHC) is currently one of the most common malignancies worldwide, occurring with high incidence and mortality. Globally, LIHC has the fourth highest mortality rate among cancer-related deaths (1). Characterized by insidious onset, high malignancy, poor prognosis, and high mortality, the highly invasive and metastatic nature of LIHC greatly threatens the outcome, prognosis, and survival of patients. Although outcomes have gradually improved with continuous progress in diagnosis and treatment, some patients with LIHC have a 5-year survival rate of only 12.1% owing to their advanced stage at the time of diagnosis (2). Immunotherapy, which is often combined with targeted therapies to prolong patient survival, is an important treatment for patients with advanced-stage LIHC who are unable to undergo surgery. Elucidating the mechanisms underlying LIHC is needed to identify new therapeutic targets for LIHC.

Necroptosis, also known as programmed necrosis, is a novel form of programmed cell death (3, 4) in which cellular self-destruction is activated by intracellular and extracellular signals when apoptosis is hindered. Necroptosis is thought to play a key role in cancer regulation, including carcinogenesis, as well as cancer metastasis, immunity, and subtypes (5, 6). Some common chemotherapeutic agents have been found to promote necroptosis and thus inhibit tumor progression (7), which may help determine the relationship between LIHC and necroptosis. A previous study induced necroptosis by hydrodynamic tail vein injection, which reshaped the tumor microenvironment (TME) and regulated hepatocellular carcinoma (HCC) subtypes through immune cell activation (6). Ferroptosis is an iron-dependent process in which lipid peroxidation products accumulate at lethal levels (8, 9). Ferroptosis and necroptosis have been identified as novel approaches to suppress tumor growth through CD8+ T cells (10), which can induce cell death or apoptosis by releasing cytokines such as IFN-γ, GzmA, and GzmB (11, 12). Enhanced anticancer immunity and tumor suppression can be achieved by modulating ferroptosis and necroptosis, suggesting potential improved outcomes for patients with HCC (13).

Long non-coding RNAs (lncRNAs) are defined as noncoding transcripts greater than 200 bp in length. lncRNAs play an important role in the development of various tumors, regulate the migration and metastasis of tumor cells, and have important value for tumor assessment and prognosis (14, 15). Studies have demonstrated that lncRNAs are novel regulators in HCC (16). Moreover, some lncRNAs have been found to induce ferroptosis and necroptosis in HCC cells (17, 18), suggesting a new therapeutic approach for HCC.

Constructing HCC prognosis diagnostic models to determine candidates suitable for immune checkpoint inhibitor immunotherapy and chemotherapy can guide individualized patient treatment plans (19). Therefore, the aim of this study was to analyze the correlation between ferroptosis- and necroptosis-related lncRNAs (F-NLRs) and the TME in LIHC using data obtained from the liver cancer database in The Cancer Genome Atlas (TCGA). We sought to reveal the potential prognostic value of a combined F-NLR signature model to help improve the outcomes of HCC treatment.

## Materials and methods

### LIHC data analysis

Data and clinical information were obtained for 377 patients with LIHC from the TCGA database, including patient age, sex, stage, and survival status.

### Identification of F-NLRs

A total of 259 ferroptosis-related genes (FRGs) and 76 necrosis-related genes (NRGs) were retrieved from the FerrDb and GeneCards databases, respectively. Ferroptosis Related LncRNAs (FRLRs) were identified by Spearman’s correlation coefficient analysis based on the expression profiles of FRGs and lncRNAs (|R| > 0.5, *P* < 0.05). NRLRs were identified by Spearman’s correlation coefficient analysis based on the expression profiles of NRGs and lncRNAs (|R| > 0.5, *P* < 0.05). F-NLRs were identified among FRLRs and Necroptosis Related LncRNAs (NRLRs) using the “clusterProfiler”, “ggplot2”, and “enrichment plots” packages in R. The biological significance of differentially expressed F-NLRs was determined *via* Gene Ontology (GO) enrichment analysis.

### Construction of F-NLR prognostic model for LIHC

Using the R package glmnet, the LASSO Cox regression technique was employed to identify F-NLRs associated with overall survival (OS) of patients with LIHC. The combined F-NLR signature was assessed as an independent prognostic factor for patients with liver cancer using univariate and multivariate Cox regression analyses. Risk value calculations were then performed using the risk assessment equation = (β1 × F-NLRs -1) + (β2 × F-NLRs -2) +… + (βn × F-NLRs -n). The prognostic characteristics of the F-NLR model were combined with independent LIHC factors obtained in the TCGA to create a hybrid nomogram. Patients with LIHC were divided into low- and high-risk groups according to the median risk score for survival difference analysis. Receiver operating characteristic curves (ROCs) were analyzed using clinical variables in hybrid nomograms and the combined F-NLR signature to predict the accuracy of 1-, 3-, and 5-year OS. An area under the curve (AUC) value > 0.70 was considered to have good prognostic value. To better understand the relationship between F-NLRs and the TME, infiltration values were calculated for the TCGA-LIHC cohort using XCELL (20), TIMER (21), QUANTISEQ (22), MCPCOUNTER (23), EPIC (24), CIBERSORT-ABS (25), and CIBERSORT(26).

### Kaplan-Meier survival (K-M) and principal component analysis (PCA)

Using the combined F-NLR signature model, the data from patients with LIHC were analyzed using K-M survival curves and PCA. The immunotherapy TIDE model was applied using the half-maximal inhibitory concentrations (IC50) listed in the Genomics of Drug Sensitivity in Cancer (GDSC) database. Levels of tumor-infiltrating immune cells were evaluated using Spearman’s correlation analysis. The treatment response was assessed to predict immunotherapy success.

### Cell culture

Normal hepatocyte cell line WRL68 and LIHC cell lines SMMC7221, Bel7405, Bel7402, and HepG2 were purchased from the Chinese Academy of Sciences and cultured separately in 1640 medium (Gibco, Grand Island, NY, USA) with 10% fetal bovine serum (AusGeneX, Molendinar, Australia) at 37°C under 5% CO2 and saturated humidity.

### Quantitative real-time polymerase chain reaction (qRT-PCR)

Total RNA was extracted from cultured cells using RNAiso Plus (Takara Bio, Beijing, China). cDNA synthesis from total RNA was performed using the PrimeScript RT kit (Takara Bio). Quantitative analysis was performed using TB Green Premix Ex Taq II (Takara Bio). The primers were synthesized by Sangon Biotech Co. Ltd. (Shanghai, China) (**Table 1**), Real-time quantitative polymerase chain reaction (qPCR) was performed using a CFX96 TouchTM Real-Time PCR instrument (Bio-Rad, Hercules, CA, USA). Each sample was analyzed in triplicate to ensure accurate quantification and averaging of threshold cycle numbers (Ct). Finally, expression levels were calculated using the 2^-ΔΔCt^ method. The thermal cycling conditions were as follows: 40 cycles of 98 °C for 30 s, 98 °C for 5 s, and 60 °C for 5 s. GAPDH served as the internal reference for normalization.

**Table 1:** Primers for qPCR.

Gene	Forward primer	Reverse primer
*AC099850.3*	3’-CAAGTAACTGGGACTACAGGTGTGC-5’	3’-CAGCCTGGGAAACATAGCGAGAC-5’
*KDM4A-AS1*	3’-GAGGCAGGAGAATGGCGTGAAC-5’	3’-TTGAGATGGAGTCTTGCTCTGTTGC-5’
*BACE1-AS*	3’-TGGCTGTTGCTGAAGAATGTGACTC-5’	3’-CAACCTTCGTTTGCCCAAGAAAGTG-5’
*ZFPM2-AS1*	3’-CAGAGGAGCGATGAAAGTGTGAGTG-5’	3’-CTGAATGCCCATAAGGGAAGGAAGG-5’
*MKLN1-AS*	3’-TGGTGGTGTTTCTCTCTGAAAGCAG-5’	3’-AGATGGCAGCGGAGTCCTCAAG-5’
*GAPDH*	3’-AGAAGGCTGGGGCTCATTTG-5’	3’-AGGGGCCATCCACAGTCTTC-5’

### Statistical analysis

The patient data were analyzed using R package version 4.0.2 R Core Team, 2020. The proportions of tumor-infiltrating immune cells were compared using the Wilcoxon test. Differences in clinical features were analyzed using the Chi-squared test. The PCR data were analyzed using the independent samples t-test with GraphPad Prism 8.0 software (GraphPad Software, La Jolla, CA, USA). A *P*-value < 0.05 was considered statistically significant.

## Results

### Identification of prognostic F-NLR features in LIHC

**Figure 1:**
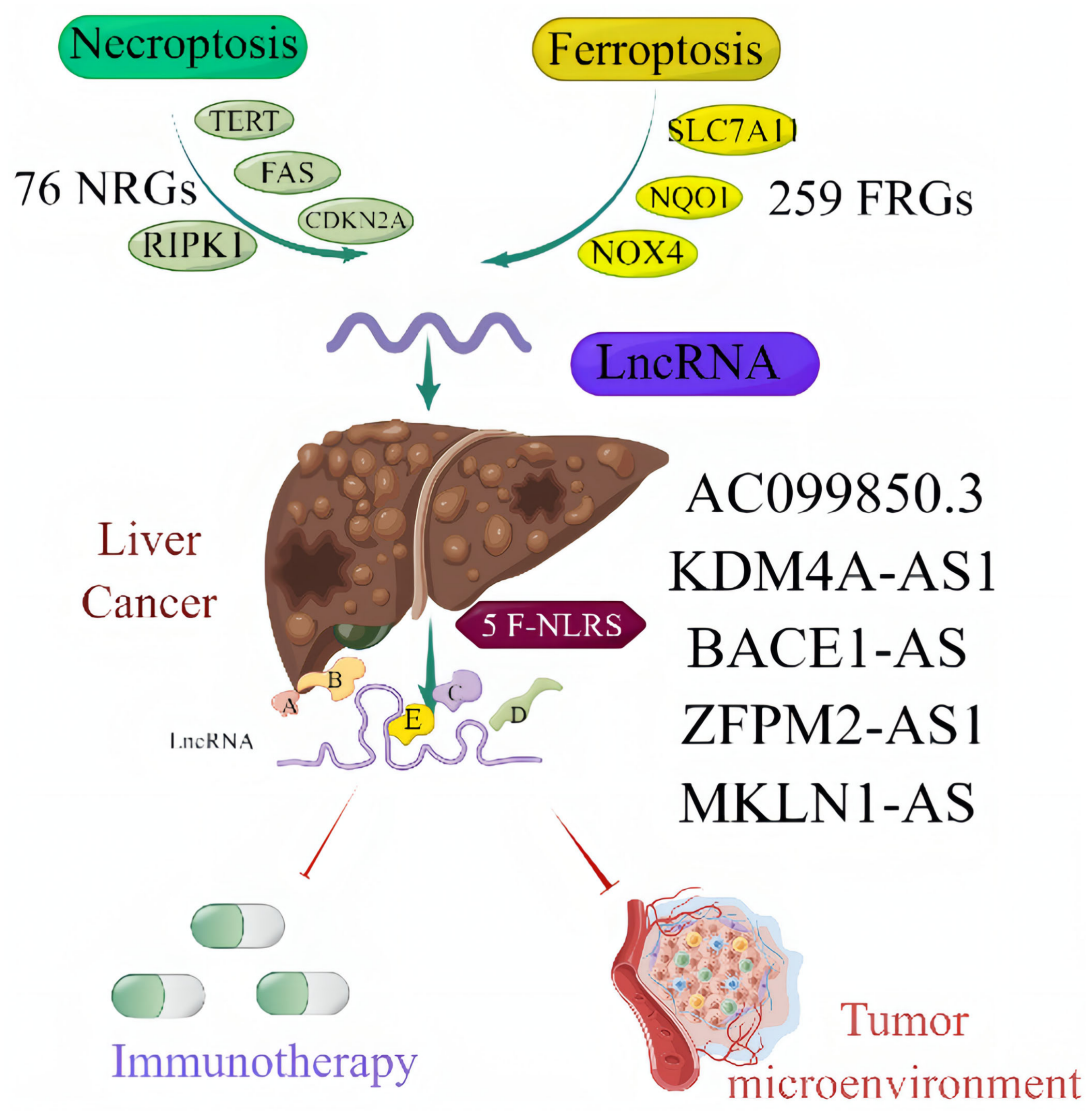
Central illustration.

**Figure 2:**
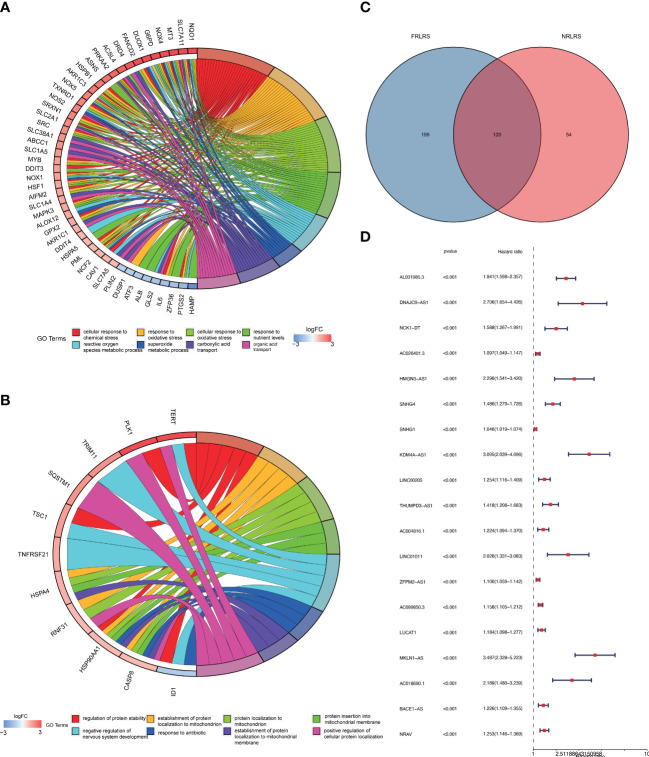
**(A)** GO enrichment analysis of ferroptosis-related genes (FRGs). **(B)** GO enrichment analysis of necroptosis-related genes (NRGs). **(C)** Venn diagram of ferroptosis-related lncRNAs (FRLRs) and necroptosis-related lncRNAs (NRLRs). **(D)** Univariate Cox regression analysis of identified lncRNAs.

**Figure 3:**
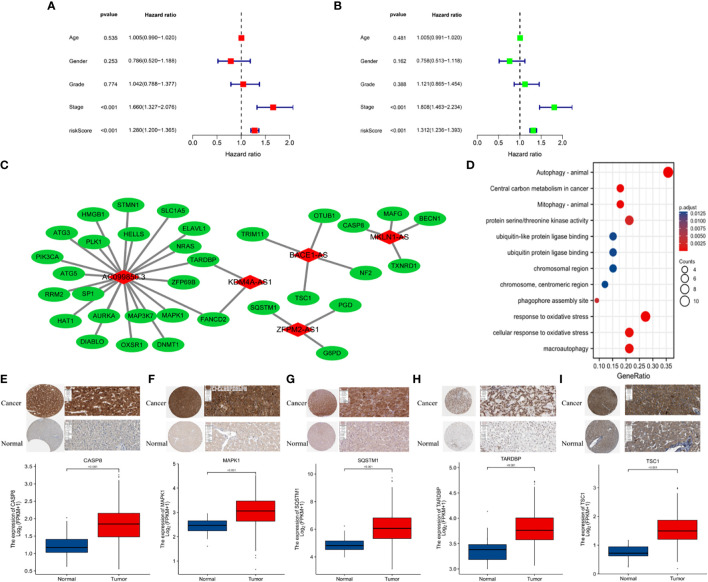
**(A)** Multivariate and **(B)** univariate Cox regression analyses for age, gender, grade, stage, and risk score. **(C)** Protein-protein interactions. **(D)** GO enrichment analysis of ferroptosis- and necroptosis-related lncRNAs (F-NLRs). **(E)** Comparison of *CASP8 TARDBP* in TCGA-LIHC tumor and normal liver tissue. **(F)** Comparison of *MAPK1* in TCGA-LIHC tumor and normal liver tissue. **(G)** Comparison of *SQSTM1* in TCGA-LIHC tumor and normal liver tissue. **(H)** Comparison of *TARDBP* in TCGA-LIHC tumor and normal liver tissue. **(I)** Comparison of *TSC1* in TCGA-LIHC tumor and normal liver tissue.

Based on data obtained from the TCGA-LIHC cohort and the literature, 319 NRLRs and 174 FRLRs were identified among 259 FRGs and 67 NRGs (**Figure 1**). **Figures 2A, B** shows the results of the GO enrichment analysis of the FRGs and NRGs. The intersection of the FRLRs and NRLRs yielded 120 F-NLRs (**Figure 2C**). Univariate Cox regression analysis showed a significant increase in the expression of 19 F-NLRs compared with that obtained by multivariate Cox analysis (**Figure 2D**). Among them, five F-NLRs were identified, including AC099850.3, KDM4A-AS1, BACE1-AS, ZFPM2-AS1, and MKLN1-AS. In the univariate Cox regression analysis, the hazard ratio (HR) and 95% confidence interval (CI) for the risk score were 1.312 and 1.236-1.393, respectively (*P* < 0.001). The HR and CI in the multivariate Cox regression analysis were 1.280 and 1.200-1.365, respectively (**Figures 3A, B**). Apart from the F-NLR prognostic factors affecting OS, stage was found to be an independent prognostic parameter (HR: 1.66, CI: 1.327-2.076, *P* < 0.001). The F-NLRs and their associated genes are shown in **Figure 3C**. The GO enrichment analysis results for the differentially expressed genes (DEGs) are shown in **Figure 3D**. The F-NLRs were mainly involved in autophagy, central carbon metabolism in cancer, mitophagy, serine- and threonine-specific protein kinase activities, ubiquitin-like protein ligase binding, ubiquitin protein ligase binding, chromosomal region, phagophore assembly site, response to oxidative stress, cellular response to oxidative stress, and macroautophagy. Based on the TCGA database, we found that the expression of real hub genes was significantly elevated in liver carcinoma compared with normal tissues. Moreover, immunohistochemistry staining obtained from The Human Protein Atlas database also demonstrated the de-regulation of real hub gene expression (**Figures 3E–I**).

**Figure 4:**
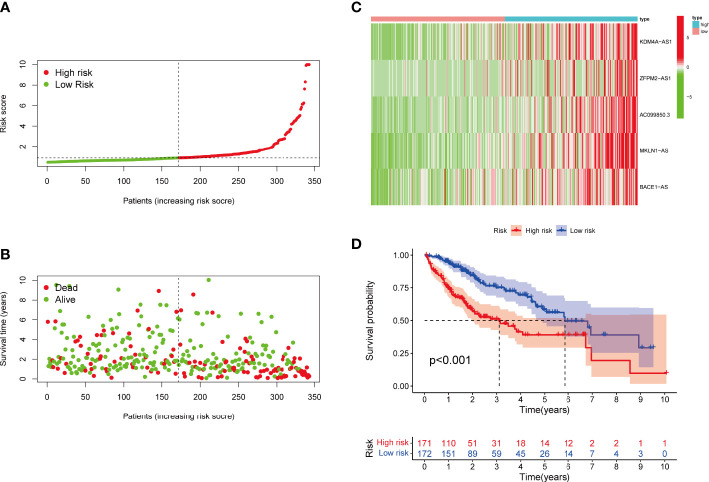
**(A)** Risk score distribution of patients with LIHC in the high- and low-risk groups. **(B)** Survival status of patients in the high- and low-risk groups. **(C)** Heat map of the expression of ferroptosis- and necroptosis-related lncRNAs (F-NLRs) in the high- and low-risk groups. **(D)** Overall survival analysis of patients in the high- and low-risk groups.

**Figure 5:**
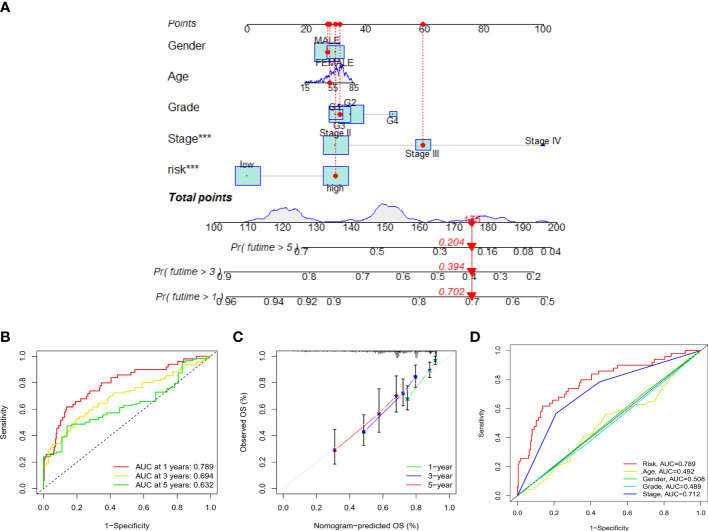
**(A)** Nomogram combining clinicopathological variables and risk scores to predict 1-, 3-, and 5-year overall survival of patients with LIHC (*** *P* < 0.001). **(B)** Accuracy of risk characteristics in predicting 1-, 3-, and 5-year characteristic curves, based on the entire set. **(C)** Calibration curves for 1, 3 and 5 years to test the agreement between actual and predicted results. **(D)** Predictive accuracy of risk models compared to clinicopathological characteristics such as risk score, age, gender, grade, and stage.

Risk level scores [Risk score = (0.537 × KDM4A-AS1) + (0.059 × ZFPM2-AS1) + (0.0924 × AC099850.3) + (0.793 × MKLN1-AS) + (0.112 × BACE1-AS)] were assigned to patients in the TCGA-LIHC cohort, enabling classification into low- and high-risk groups for prognostic analysis (**Figure 4A**). The scatter plot combining sample data with clinical information was constructed to demonstrate the patient survival statistics for both risk groups (**Figure 4B**). Five DEGs among the F-NLRs were screened (**Figure 4C**). OS was longer for patients in the low-risk group than in the high-risk group (*P* < 0.001) (**Figure 4D**). Mortality was higher for patients in the high-risk group than in the low-risk group (*P* < 0.001). A hybrid nomogram for LIHC was constructed by combining the five F-NLR prognostic features with independent factors (**Figure 5A**). Patients were scored according to the independent factors and their survival was predicted. Time-dependent ROC analysis was used to assess the sensitivity and specificity of the prognostic model. The prognostic AUC values were 0.789, 0.694, and 0.632 at 1, 3, and 5 years, respectively (**Figure 5B**). **Figure 5C** shows the calibration plot of the column line graph. Risk score (0.789) and stage (0.712) were the main predictors of the risk model score (**Figure 5D**), suggesting that the combined F-NLR signature was superior to traditional clinical variables for predicting the outcomes of patients with liver cancer.

### Survival analysis and PCA

**Figure 6:**
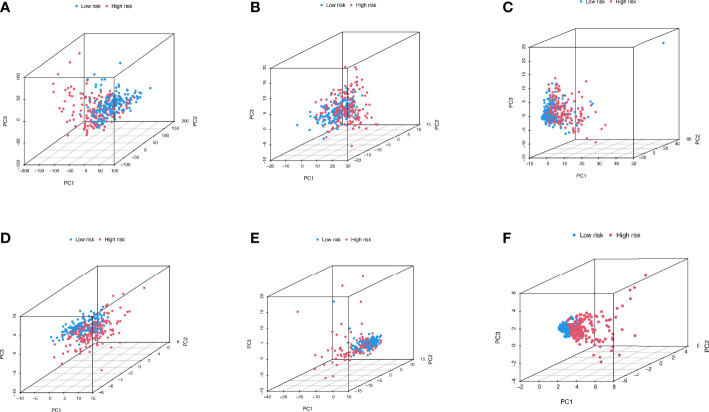
PCA dimensionality reduction analysis distinguishing between high- and low- risk groups for **(A)** entire gene expression in LIHC cohort; **(B)** 259 ferroptosis-related genes in high- and low-risk groups; **(C)** 174 ferroptosis-related lncRNAs (FRLRs) in high- and low-risk groups; **(D)** 67 necroptosis-related genes in high- and low-risk groups; **(E)** 319 necroptosis-related lncRNAs (NRLRs) in high- and low-risk groups; **(F)** Five ferroptosis- and necroptosis-related lncRNAs (F-NLRs) in high- and low-risk groups.

**Figure 7:**
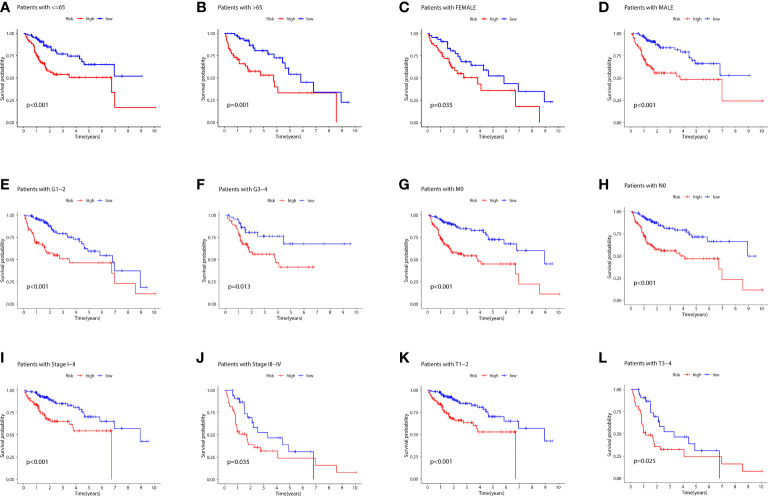
**(A)** K-M curves for age ≤ 65 for the different risk groups of LIHC patients. **(B)** K-M curves for age > 65 years for the different risk groups of LIHC patients. **(C)** K-M curves for female for the different risk groups of LIHC patients. **(D)** K-M curves for male for the different risk groups of LIHC patients. **(E)** K-M curves for stages G1-2 for the different risk groups of LIHC patients. **(F)** K-M curves for stages G3-4 for the different risk groups of LIHC patients. **(G)** K-M curves for stage M0 for the different risk groups of LIHC patients. **(H)** K-M curves for stage N0 for the different risk groups of LIHC patients. **(I)** K-M curves for stages I-II for the different risk groups of LIHC patients.**(J)** K-M curves for stages III-IV for the different risk groups of LIHC patients. **(K)** K-M curves for stages T1-2 for the different risk groups of LIHC patients. **(L)** K-M curves for stages T3-4 for the different risk groups of LIHC patients.

**Figure 6** depicts the PCA dimensionality reduction analysis plotted to classify patients with LIHC into the high- and low-risk groups. The expression levels of all LIHC-associated genes (**Figure 6A**) in spatial stereo were not clearly distinguished between the high- and low-risk groups. Additionally, FRG and FRLR expression levels were not well distinguished between the risk groups (**Figures 6B, C**). Similarly, NRG and NRLR expression levels were not well distinguished between the risk groups (**Figures 6D, E**). However, analysis of F-NLR expression levels revealed a clear distinction between the high- and low-risk groups (**Figure 6F**). The OS of patients with LIHC was evaluated using K-M curves, stratifying the data according to age, sex, stage, grade, lymph node, and metastasis, as shown in **Figures 7A–L**. Patients in the low-risk group had longer OS than those in the high-risk group (*P* < 0.001).

### IC50 assessment of treatment response and mutation data analysis

**Figure 8:**
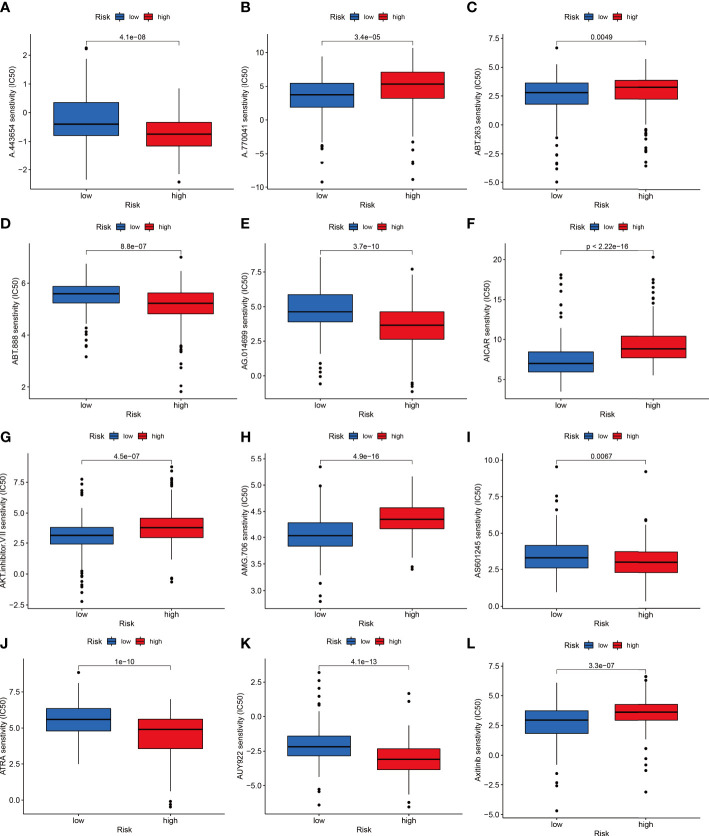
The low-risk group is shown in blue on the abscissa, and the high-risk group is shown in red. The IC50 value of drug target sensitivity is shown on the ordinate. **(A)** A.443654. **(B)** A.770041. **(C)** ABT.263. **(D)** ABT.888. **(E)** AG.014699. **(F)** AICAR. **(G)** AKT inhibitor VIII. **(H)** AMG.706. **(I)** AS601245. **(J)** ATRA. **(K)** AUY922. **(L)** Axitinib.

**Figure 9:**
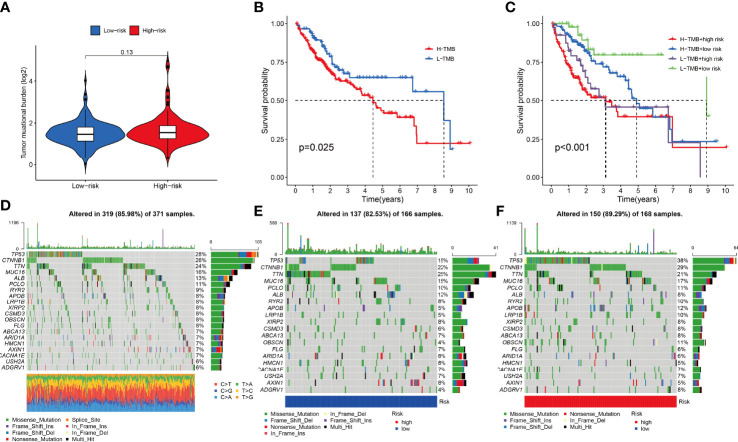
**(A)** Combined F-NLR signature model to assess the expression of tumor mutational burden (TMB) in high- and low-risk groups. **(B, C)** Survival analysis of TMB in the TCGA-LIHC cohort. **(C)** Survival analysis of TMB with risk group in the TCGA-LIHC cohort. **(D)** Gene mutation information of the TCGA-LIHC cohort. **(E)** Gene mutations information of patients in the low-risk group. **(F)** Gene mutation information of patients in the high-risk group.

The combined F-NLR signature model was employed to evaluate potential candidate drugs for LIHC. Patients in the high-risk group and low-risk group were significantly more sensitive to 12 selected immunotherapeutic agents with significantly different IC50 values (**Figures 8A–L**). According to the assessed IC50 values, patients in the low-risk group were more sensitive to six drugs, including A.77004, ABT.263, AICAR, AMG706, Axitinib, and AKT inhibitor VIII, whereas patients in the high-risk group were more sensitive to ABT.888, A.443654, AG.014699, ATRA, AS601245, and AUY922. The immunotherapy outcomes of the LIHC cohort in the TCGA database were assessed using the F-NLR model. As shown in **Figure 9A**, the outcomes of patients in the high- and low-risk groups did not differ significantly, indicating that the F-NLR classifier index did not correlate well with tumor mutational burden (TMB). Low TMB in LIHC was associated with high survival, and patients in the low-risk group with low TMB had the highest survival rate (**Figures 9B, C**). **Figure 9D** shows the gene mutation information of the TCGA-LIHC cohort, while **Figures 9E, F** shows the mutation information for 20 commonly mutated genes in LIHC in the high- and low-risk groups.

**Figure 10:**
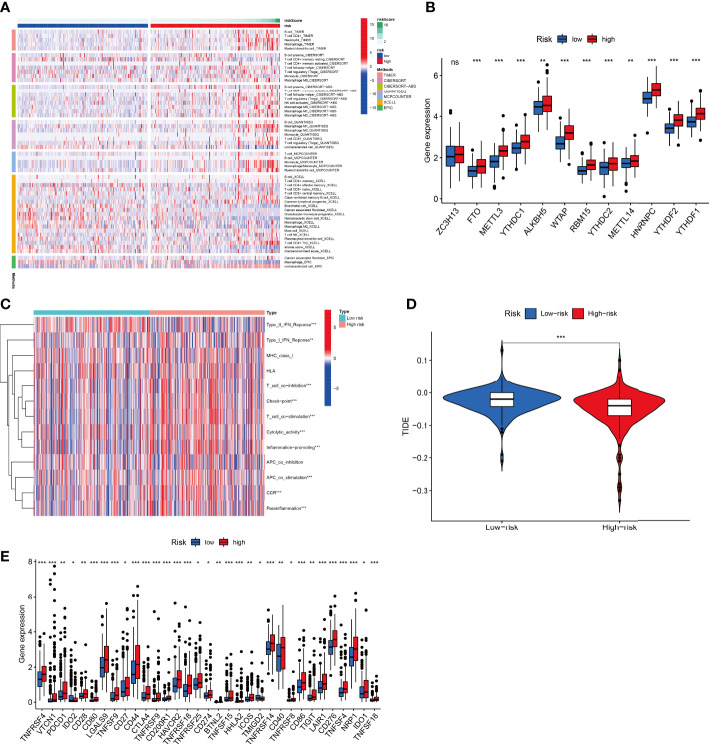
Combined F-NLR signature model to predict immunotherapy responses. **(A)** Heatmap of immune responses in the high- and low-risk groups based on different algorithms. **(B)** Expression of m^6^A-related genes in the high- and low-risk groups. **(C)** Single-sample gene set enrichment analysis of the association between immune cell subpopulations and related functions in the high- and low-risk groups. **(D)** TIDE model results for the high- and low-risk groups. **(E)** Expression of immune checkpoints in the high- and low-risk groups. (*P < 0.05, **P  < 0.01, ***P  < 0.001.).

Multiple enrichment analysis algorithms, including TIMER, MCPCounter, GSEA, and XCELL, were used to produce a heatmap of immune responses (**Figure 10A**). Considering the importance of checkpoint inhibitor immunotherapy, differences in immune checkpoint gene expression were assessed between the two risk groups. The expression of almost all N^6^-methyladenosine (m^6^A) related genes differed between the two risk groups, except ZC3H13 (**Figure 10B**). Correlation analysis between immune cell subpopulations and their functions revealed that T cell functions, including type-II and type-I IFN responses, checkpoints, T cell co-inhibition, T cell co-stimulation, cytolytic activities, inflammation promotion, antigen-presenting cell co-stimulation, chimeric co-stimulatory receptors, and para-inflammation, differed between the risk groups (**Figure 10C**). The TIDE algorithm predicted that patients in the low-risk group would respond better to immunotherapy (**Figure 10D**). Furthermore, patients in the high-risk group showed better activation at most immune checkpoints than those in the low-risk group (**Figure 10E**).

### Verification of F-NLR by qPCR

**Figure 11:**
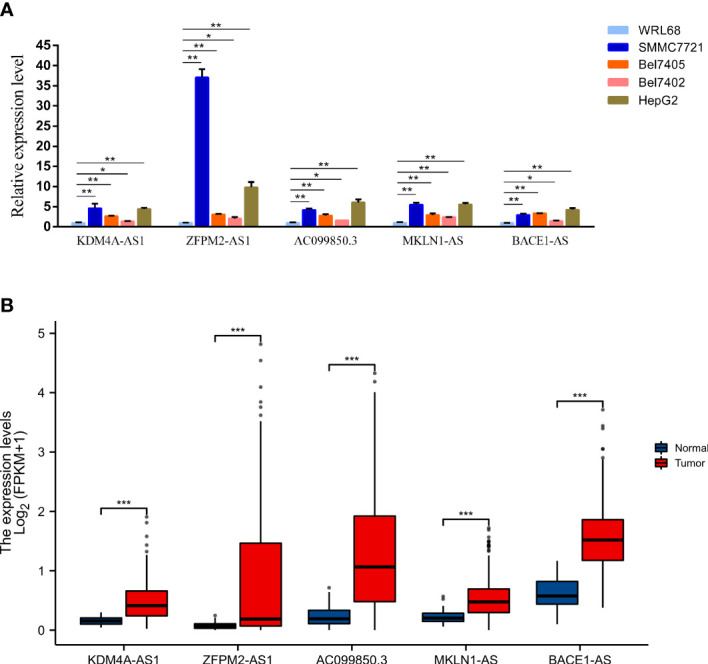
**(A)** Levels of proteins encoded by the five ferroptosis- and necroptosis-related lncRNAs (F-NLRs) in normal hepatocytes and LIHC cell lines, as determined by qRT-PCR. **(B)** Expression of proteins encoded by the five F-NLRs in the TCGA-LIHC cohort. (*P < 0.05, **P  < 0.01, ***P  < 0.001).

**Figure 12:**
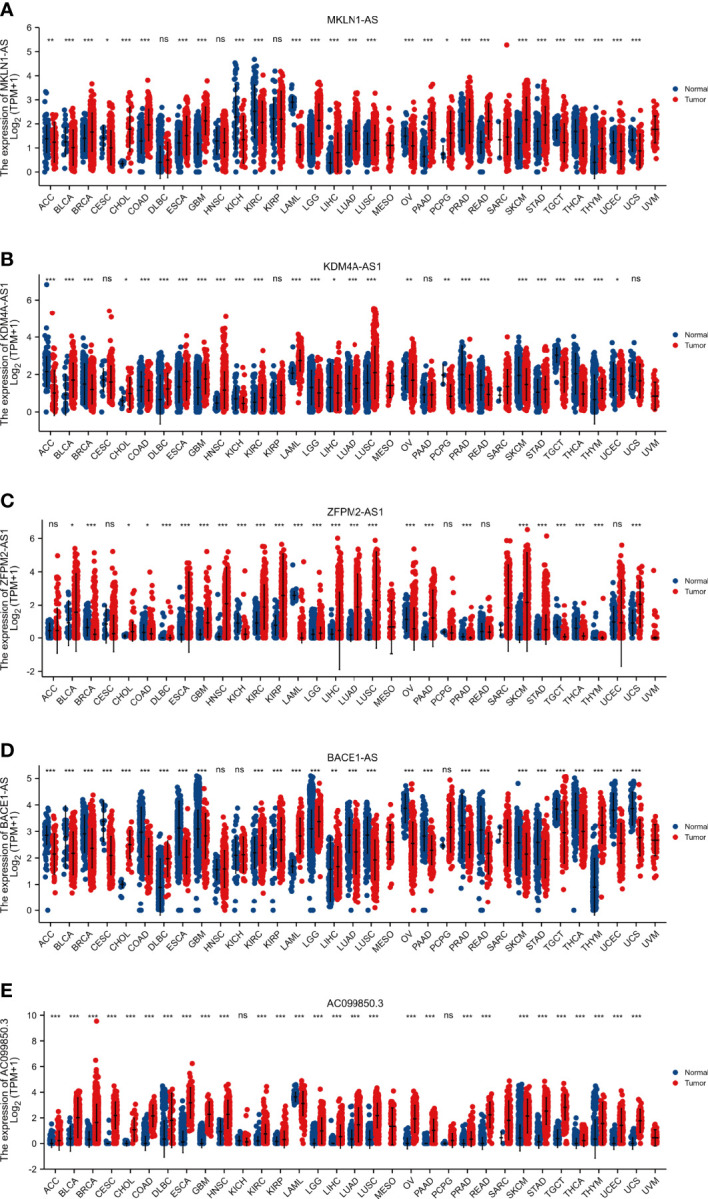
Pan-cancer analysis of the expression of five ferroptosis- and necroptosis-related lncRNAs. (*P < 0.05, **P < 0.01, ***P < 0.001).

The expression levels of five key F-NLRs were analyzed using qPCR in this study (**Figure 11A**). *KDM4A-AS1*, *ZFPM2-AS1*, *AC099850.3*, *MKLN1-AS*, and *BACE1-AS* RNA levels were significantly higher in LIHC cell lines than in the normal hepatocyte line WRL68. *ZFPM2-AS1* was significantly elevated in all LIHC cell lines (*P* < 0.05) and was ten times higher in SMMC7721 and HepG2 cells compared to WRL68 cells. RNA levels of KDM4A-AS1, *AC099850.3*, *MKLN1-AS*, and *BACE1-AS* were also significantly elevated (*P* < 0.05), but not as high as levels of *ZFPM2-AS1*. Interestingly, *AC099850.3* RNA levels in all LIHC cell lines were higher than those in WRL68 cells. Bel7405 cells had lower RNA levels of the five F-NLRs compared to other LIHC cell lines. The RNA expression levels of the five F-NLRs in the qPCR results were consistent with the TCGA-LIHC cohort data (**Figure 11B**). Pan-cancer analysis of expression levels of the five F-NLRs showed differences between most tumor types and normal tissues (**Figures 12A–E**).

## Discussion

As two novel cell death modalities, mounting evidence has demonstrated that ferroptosis and necroptosis are intimately linked with tumor progression (27–31). However, few studies have thoroughly explored the potential value of combining differentially expressed FRLRs and NRLRs as a prognostic signature of LIH to predict immunotherapy responses. With the rapid popularization of artificial intelligence (AI) technology, it has shown a broad application prospect in the medical field. AI based on machine learning technology can extract data features from a large amount of data, and build a risk stratification model of cancer patients more accurately, thus assisting doctors in clinical decision-making (32). Therefore, with the help of machine learning technology, it brings unprecedented opportunities for tumor research and diagnosis by collecting and mining available tumor data to discover the internal relations and rules (33). Machine learning is an interdisciplinary subject, especially the statistical analysis of clinical medicine, which has become a research focus. Researchers believe that LASSO is a branch of regression analysis in machine learning. Of course, the application of LASSO algorithm, as a feature screening algorithm in machine learning, will be more appropriate.

Dysfunction during necroptosis, a process is regulated by lncRNAs, is involved in the development of HCC (34–36). Thus, this study first identified lncRNAs associated with ferroptosis and necroptosis and investigated their value in LIHC immunotherapy (37, 38). The expression and prognostic significance of 319 FRLRs and 174 NRLRs in LIHC were explored. Taking these findings into consideration, a prognostic gene expression signature was constructed based on ferroptosis and necroptosis in LIHC. A total of 120 F-NLRs were involved in the regulation of protein stability, cellular protein localization, neuronal death, CD40 receptor complex, the cytoplasmic side of the plasma membrane, ubiquitin-like protein ligase binding, and ubiquitin protein ligase binding. These genes were closely linked to necrotic apoptosis and the immune response.

Among the F-NLRs identified in the study, five lncRNAs were significantly associated with OS in LIHC, including KDM4A-AS1, ZFPM2-AS1, AC099850.3, MKLN1-AS, and BACE1-AS. The expression of proteins encoded by these genes was further validated by qRT-PCR, demonstrating that all five F-NLRs were overexpressed in liver cancer cells. Expression levels of KDM4A-AS1 were significantly elevated in both LIHC tissues and cell lines, and high KDM4A-AS1 expression was associated with advanced TNM staging and lymphatic metastasis. A previous study reported that KDM4A-AS1 expression was closely linked to OS and prognosis of LIHC. Thus, KDM4A-AS1 is considered to be an important prognostic factor for patients with HCC (39). Additionally, KDM4A-AS1 has been shown to promote proliferation, migration, invasion, and epithelial–mesenchymal transition in HCC cells under loss- and gain-of-function conditions. Specifically, KDM4A-AS1 acted *via* the miR-411-5p/KPNA2/AKT pathway to promote the growth and metastasis of HCC (40). Moreover, KDM4A-AS1 was shown to play an important role in the progression of castration-resistant prostate cancer (CRPC) and enzalutamide resistance by regulating the deubiquitination of androgen receptors (AR) and AR splice variants, thus providing a potential therapeutic target for CRPC (41). These findings suggested that KDM4A-AS1 may be an independent potential prognostic biomarker for patients with LIHC. Furthermore, KDM4A-AS1 may be important for controlling the occurrence of LIHC since it is closely associated with the TME. However, the role of KDM4A-AS1 in carcinogenesis remains unclear. Meanwhile, the expression of ZFPM2-AS1 was upregulated in HCC and high ZFPM2-AS1 expression was associated with age, T stage, and pathological stage of the patient. A previous study reported that STAT1 activated the translational expression of ZFPM2-AS1 in HCC, thus modulating levels of the protein (42). Knockdown of ZFPM2-AS1 inhibited cell proliferation, migration, invasion, and apoptosis (43). Furthermore, ZFPM2-AS1 overexpression predicted poor prognosis in lung adenocarcinoma (LUAD) and boosted cell proliferation in these tumors (44). Previous studies have indicated that AC099850.3 plays a role in cancer. A study by Zhou demonstrated that AC099850.3 was associated with prognosis and competition between endogenous RNAs in tongue squamous cell carcinoma (45). AC099850.3 was also significantly upregulated in non-small cell lung cancer cells, and its depletion markedly inhibited cell proliferation and migration in LUAD cells (46). Because AC099850.3 is significantly associated with the TME in HCC, it may provide a potential immunotherapy target (47). Research demonstrated that MKLN1-AS upregulation was associated with vascular invasion, suggesting that MKLN1-AS was involved in tumor progression (48). As a competitive endogenous RNA, MKLN1-AS increased the expression of hepatoma-derived growth factor in HCC cells by competitively binding to miR-654-3p (49). Patients with HCC may benefit from the use of MKLN1-AS as a therapeutic target in the future. Finally, BACE1-AS is considered to be an effective biomarker for predicting the prognosis of patients with LIHC and has been used in a risk model to assess LIHC prognosis (39). M2 macrophage abundance was negatively correlated with BACE1-AS levels in tenosynovial giant cell tumors, LIHC, lung squamous cell carcinoma, colon adenocarcinoma, prostate adenocarcinoma, and KIRC, and was positively correlated with BACE1-AS levels in thyroid carcinoma, cervical squamous cell carcinoma and endocervical adenocarcinoma, and acute myeloid leukemia. The BACE1-AS protein may promote tumor antigen presentation in cancer cells, thereby suppressing immune responses (50). Moreover, BACE1-AS may inhibit the proliferation and invasion of human ovarian cancer stem cells, providing a potential new mechanism for anisomycin treatment in LIHC (51).

In the present study, patients with LIHC were divided into low- and high-risk groups according to the median risk score, revealing that patients in the low-risk group had better outcomes than those in the high-risk group. Risk score and stage were independent predictors of OS in patients with LIHC. Further, survival prediction for patients with LIHC was improved using the combined F-NLR signature model compared to that achieved using conventional clinical characteristics. An AUC value closer to 1.0 indicates a more accurate prediction of diagnosis, with an AUC value > 0.7 indicating a highly accurate model. High statistical significance was established for OS at 1, 3, and 5 years, suggesting that the combined F-NLR signature can accurately predict the prognosis of patients with LIHC.

Subsequently, potential candidate drugs for LIHC were identified using the F-NLR model. Assessment of IC50 values, the TME, and immunotherapy responses indicated that patients in the high-risk group responded more positively to immunotherapeutic agents. TMB scores were calculated using the cellular mutation data from the TCGA-LIHC cohort, and the low-risk group did not outperform the high-risk group. A high TMB score in LIHC was associated with poorer outcome and could be used as a prognostic marker. The results indicated that the combined F-NLR signature model had a higher prognostic value than TMB. KDM4A-AS1, ZFPM2-AS1, AC099850.3, MKLN1-AS, and BACE1-AS may provide biomarkers for drug therapy at different tumor stages and gene mutation loads, thus facilitating precise treatment for patients with LIHC. However, the mechanisms of these five lncRNAs in liver cancer warrant further exploration.

According to the combined F-NLR signature model, patients in the low- and high-risk groups displayed great differences in drug sensitivity, which were associated with necroptosis and the TME. These results suggest that patients with LIHC who differentially express F-NLRs may have different drug sensitivities for different targets, reflecting individual differences in treatment responses. These results are relevant for future LIHC research investigating therapeutic targets related to immunotherapy and the TME. Due to differences in immune responses, targeted therapy can be combined with immunotherapy to provide personalized and precise treatment for patients with LIHC based on lncRNA expression levels. Although KDM4A-AS1, ZFPM2-AS1, AC099850.3, MKLN1-AS, and BACE1-AS were highly expressed in LIHC cell lines, their expression differed between most tumor types and normal tissues. In this study, we revealed the differential expression of five lncRNA as survival predictors in 33 cancers based on Ferroptosis and Necroptosis. Our results show that except BACE 1-AS in 5 F-NLRS, the above-mentioned lncRNA is obviously up-regulated in many cancers, especially in solid tumors, and its high expression can be used as a prognostic indicator. This is also the first lncRNA pan-cancer analysis based on Ferroptosis and Necroptosis, which aims to comprehensively studying its potential role in potential tumor prognosis and tumor treatment.

Nevertheless, this study had some limitations. Firstly, there is a possibility of overfitting, which occurs when the model is too complex and too close to the training data, leading to poor performance on new data. Secondly, there may be biases in the selection of training and validation datasets. Although publicly available datasets were used in this study, these datasets may not represent a broader population of LIHC patients. Additionally, the relatively small sample size of this study may limit the generalizability of the results. Thirdly, there is a lack of experimental validation for more F-NLRs. Although we did confirm differential expression of F-NLRs in HCC cell lines and normal liver cells, further experiments are needed to investigate the roles of these lncRNAs in ferroptosis and necroptosis. Finally, our study only focused on F-NLRs and did not consider other potential biomarkers or clinical variables that may be important for predicting HCC prognosis and immunotherapy response. Despite these limitations, this study provides valuable insights into the potential role of F-NLRs in HCC and may guide future research in this field.

## Conclusions

We have identified lncrna related to iron death and necrosis and apoptosis by machine learning, and studied their potential value in LIHC immunotherapy. According to several analytical models, we verified 120 F-NLRs, and finally determined that 5 F-NLRs were prognostic predictors of TCGA-LIHC. After further qPCR verification, it was found that these five molecules had been over-expressed in hepatocellular carcinoma cell line, which was consistent with the expression trend of bioinformatics analysis. The five F-NLRs (KDM4A-AS1, ZFPM2-AS1, AC099850.3, MKLN1-AS, and BACE1-AS) identified in the current study provide a predictive signature representing ferroptosis and necroptosis in LIHC, which correlated well with patient prognosis, clinicopathological characteristics, and immunotherapy responses. We have identified lncrna related to iron death and necrosis and apoptosis by machine learning, and studied their potential value in LIHC immunotherapy. According to several analytical models, we verified 120 F-NLRs, and finally determined that 5 F-NLRs were prognostic predictors of TCGA-LIHC. After further qPCR verification, it was found that these five molecules had been over-expressed in hepatocellular carcinoma cell line, which was consistent with the expression trend of bioinformatics analysis. The five F-NLRs (KDM4A-AS1, ZFPM2-AS1, AC099850.3, MKLN1-AS, and BACE1-AS) identified in the current study provide a predictive signature representing ferroptosis and necroptosis in LIHC, which correlated well with patient prognosis, clinicopathological characteristics, and immunotherapy responses. The clinical implications of our findings are twofold. Firstly, our LncRNA signature may serve as a useful prognostic biomarker for liver cancer patients, providing clinicians with a tool to better predict patient outcomes and inform treatment decisions. Secondly, our signature may have implications for the use of immunotherapy in LIHC.

## Data availability statement

The original contributions presented in the study are included in the article/supplementary material. Further inquiries can be directed to the corresponding authors.

## Author contributions

Conceptualization, JX and ZB. Data curation, LZ and ZY. Formal analysis, ZB. Methodology, JX. Resources, JX. Software, LZ. Supervision, JX and ZB. Validation, ZY. Writing – original draft, LZ. Writing – review and editing, JX. Project administration, JX. Funding acquisition, JX and ZB. All authors contributed to the article and approved the submitted version.
